# Early detection of opioid-induced constipation in patients initiating weak opioids for chronic non-cancer pain

**DOI:** 10.1038/s41598-026-45169-x

**Published:** 2026-03-30

**Authors:** Yasuhide Morioka, Akira Hashimoto, Yuichi Koretaka, Shihomi Wada, Motoki Sonohata, Asbjørn Mohr Drewes

**Affiliations:** 1 Medical Affairs Department, Shionogi & Co., Ltd., GRAND GREEN OSAKA South Building Park Tower, 5-54 Ofuka-cho, Kita-ku, Osaka, 530-0011 Japan; 2 Department of Orthopaedic Surgery, Japan Community Health Care Organization (JCHO) Saga Central Hospital, Saga, Japan; 3 Data Science Department, Shionogi & Co., Ltd., Osaka, Japan; 4 Mech-Sense & Center for Pancreatic Diseases, Department of Gastroenterology and Hepatology, Aalborg University Hospital, Aalborg, Denmark; 5 Clinical Institute, Aalborg University, Forskningens Hus, Aalborg, Denmark

**Keywords:** Non-cancer pain, Opioid-induced constipation, Positive likelihood ratio, Posterior probabilities, Rome IV diagnostic criteria, Weak opioids, Cancer, Gastroenterology, Health care, Medical research

## Abstract

This post hoc analysis of a Japanese cohort study aimed to identify factors that could assist in early prediction of opioid-induced constipation (OIC). The onset of OIC and the predictive accuracy (sensitivity, specificity, likelihood ratios, and positive predictive values) of early constipation symptoms for OIC onset were assessed within 6 days of weak opioid initiation. Of 63 patients (≥ 18 years) without prior constipation who initiated weak opioids for chronic musculoskeletal pain, 23.8% of them met the Rome IV diagnostic criteria for OIC onset after 3 days. Straining (34.9%), incomplete evacuation (25.4%), and lumpy/hard stools (15.9%) were the common symptoms, with the highest positive likelihood ratio of 2.4 for lumpy/hard stools. Decreased defecation frequency is commonly used for the diagnosis of OIC; however, it was reported in only 1.6% of patients at day 3. The positive predictive value of developing OIC by day 14, based on the early symptoms at day 3, was 70.0% for lumpy/hard stools, 59.1% for straining, 56.3% for sensation of incomplete evacuation, and 72.7% for self-awareness of constipation. Early observation of symptoms such as lumpy/hard stools and self-awareness of constipation could be useful for early prediction of the risk of developing OIC after initiation of weak opioids.

## Introduction

Opioids are recommended as third-line treatment after non-pharmacological procedures and non-opioid analgesics for managing chronic non-cancer pain in Japan^1^. Despite their effectiveness, they are associated with gastrointestinal side effects such as nausea, vomiting, gastroesophageal reflux, and constipation^2,3^. Among these, opioid-induced constipation (OIC) is of particular concern due to its reported occurrence in 41%-57% of patients receiving opioids for chronic non-cancer pain^2^. A web-based survey in Japan also reported that 28% of patients with chronic non-cancer pain who were using opioids experienced constipation, as based on the Rome IV diagnostic criteria for constipation^4^. Evidence suggests that tolerance does not develop to constipation with the use of opioids in contrast to symptoms such as nausea and vomiting^5–8^. Although OIC is mainly described for strong opioids, a non-clinical study has shown dose-related inhibition of the gastrointestinal motility after tramadol administration^9^. In addition, a randomized trial in healthy participants^10^ and cohort studies in patients with chronic non-cancer pain^4,11^ have reported that OIC and constipation symptoms develop after initiating weak opioids. The adverse impact of OIC on health-related quality of life was also demonstrated in patients with chronic non-cancer pain with weak opioids^12^. Therefore, careful monitoring is essential when initiating weak opioids for pain management.

The diagnosis of OIC employs the Rome IV diagnostic criteria, which are defined as the presence of at least two of the following symptoms after initiation of opioids: straining, lumpy/hard stools, sensation of incomplete evacuation, sensation of anorectal blockage, manual maneuvers to facilitate defecation, and < 3 spontaneous bowel movements per week^13,14^. The Rome IV diagnostic criteria showed 81.9% accuracy when compared with clinician assessment in patients with cancer pain^15^. Using these criteria, an internet-based survey in Japan reported OIC in 30% of patients (*n* = 500) with chronic non-cancer pain initiating opioids for at least 3 months; the majority of them (89%) were receiving weak opioids^4^. In our recent observational study, including 63 patients who were newly prescribed weak opioids for chronic non-cancer pain, we reported a cumulative incidence of OIC of 30.2% in the first week, which increased to 49.2% by the end of the second week^11^. These findings indicate that patients develop constipation soon after opioid initiation. Since the Rome IV criteria require symptoms to be assessed for more than 1 week, we aimed to assess OIC onset and the predictive accuracy of early constipation symptoms for OIC. In the present study, we focused on the early symptom onset based on patient-reported outcomes. As these symptoms do not require physician examination or clinical testing, patients can track them independently every day, allowing precise reporting of the timing and progression of symptom onset, facilitating the collection of data.

## Methods

### Study design and data source

This post hoc analysis of an observational study (UMIN000050203) was conducted in Japan to assess the incidence of OIC in patients with chronic musculoskeletal pain where weak opioids were prescribed. Patients were recruited from 17 orthopedic clinics or hospitals, and the data were collected using a web-based, patient-reported questionnaire survey. Prospective participants were provided with a written study information sheet at clinics and hospitals and requested to voluntarily complete an informed consent form via QR codes or a URL after discharge. The treating physicians were not informed about the participation of patients in this survey. Laxatives were prescribed according to routine clinical practice. The study methodology has been published in detail previously^11^.

### Study population

Inclusion criteria were: (a) patients aged ≥ 18 years who were newly prescribed weak opioids for chronic non-cancer musculoskeletal pain at the time of enrollment or within the previous day; (b) those for whom treatment duration lasted a minimum of 2 weeks; (c) those who experienced at least 3 bowel movements per week before the enrollment day; and (d) patients who were able to complete the online questionnaire using smartphones or tablets.

Exclusion criteria were patients who: (a) were hospitalized; (b) had malignant tumors at the time of enrollment; (c) received opioid treatment within the 4 weeks before enrollment; (d) fulfilled two or more of the constipation symptoms based on the Rome IV diagnostic criteria for OIC within the week before enrollment; or (e) used laxatives at the initiation of opioid therapy.

### Assessments

The prevalence of constipation symptoms on day 14 was evaluated based on Rome IV diagnostic criteria for OIC^13,14^. At a daily basis, each symptom was evaluated daily from day 1 to day 6 following opioid initiation based on the Rome IV diagnostic criteria for OIC with some modification. Straining: straining during more than 25% of defecations; lumpy or hard stools: lumpy or hard stools (Bristol Stool Form Scale 1-2) for more than 25% of defecations; sensation of incomplete evacuation: sensation of incomplete evacuation for more than 25% of defecations; sensation of anorectal obstruction/blockage: sensation of anorectal obstruction/blockage for more than 25% of defecations; manual maneuvers: manual maneuvers to facilitate more than 25% of defecations. All defecations that occurred from the initiation of opioid use to each evaluation day were included (number of defecations with each constipation symptom from the initiation of opioid use to each evaluation day/total number of defecations from the initiation of opioid use to each evaluation day). Decreased defecation frequency: to approximate the Rome IV criterion of < 3 spontaneous bowel movements per week within a partial‑week window, we modified and defined decreased defecation frequency as fewer than one bowel movement (no defecation) from day 1 to day 3 or fewer than two bowel movements from day 4 to day 6. The incidence of OIC was evaluated daily from day 1 to day 6, based on the presence of at least two of the constipation symptoms.

Self-awareness of constipation was defined as an affirmative response to feeling constipated at least once from day 1 and onward, as reported in a previous study^11^.

### Statistical analysis

All analyses were conducted using SAS 9.4 (SAS Institute, Cary, NC). The cases of OIC development (*n* = 31) or non-development (*n* = 32) by 2 weeks after initiating opioids, along with the incidence rate of OIC, were based on data from a previous study^11^.

Data on the prevalence of constipation symptoms were analyzed using descriptive statistics and are presented as frequency (n) and proportion (%). The predictive accuracy of OIC onset by day 14 was evaluated using sensitivity, specificity, and positive and negative likelihood ratios (LR+ and LR-). Positive predictive values (PPV) were also reported. These parameters were calculated based on the number of patients who developed constipation symptoms and those who reported self-awareness of constipation among patients who either developed OIC or did not by day 14 in a previous study^11^. Sensitivity, specificity, LR+, and LR- were presented with 95% confidence intervals (CIs), whereas PPV were presented as percentages.

### Ethical considerations

This was a post hoc analysis of an observational study in Japan. The study was conducted in accordance with the ethical principles based on the Declaration of Helsinki and in compliance with the “Ethical Guidelines for Life Science and Medical Research Involving Human Subjects” and the “Guidance,” as well as applicable Japanese laws and regulations. The study was approved by the Takahashi Clinic Ethics Committee of Takahashi Clinic Medical Corporation (trial registration number: UMIN000050203).

## Results

### Patient disposition and baseline characteristics

The details of patient disposition and baseline characteristics have been published previously^11^. Sixty-three patients met the eligibility criteria and were included. The baseline characteristics of included patients are provided in Table 1. The mean age of the patients was 53.1 years, with 52.4% being female. The most common sites of pain were the back and lower back (36.5%), followed by the lower limbs (30.2%). Most patients (61.9%) were prescribed tramadol hydrochloride extended-release (twice a day), followed by tramadol hydrochloride immediate-release (25.4%). The mean duration of weak opioid intake was 6.6 days, with a mean tramadol-equivalent dose of 67.2 mg/day.

**Table 1:** Baseline characteristics of included patients.

Characteristic	Patients (*n* = 63)
Age (years), mean ± SD	53.1 ± 11.4
*Sex, n (%)*	
Male	30 (47.6)
Female	33 (52.4)
*Location of pain, n (%)*	
Back and lower back	23 (36.5)
Lower limbs	19 (30.2)
Upper limb	14 (22.2)
Neck	6 (9.5)
Other areas	1 (1.6)
*Opioid, n (%)*	
Tramadol hydrochloride extended-release (twice a day)	39 (61.9)
Tramadol hydrochloride immediate-release	16 (25.4)
Tramadol hydrochloride/acetaminophen combination	8 (12.7)
Number of days of weak opioid intake (days/week), mean ± SD	6.6 ± 1.1
Tramadol-equivalent dose (mg/day), mean ± SD	67.2 ± 34.1

SD, standard deviation.

### OIC onset, constipation symptoms, and self-awareness of constipation

**Fig. 1:**
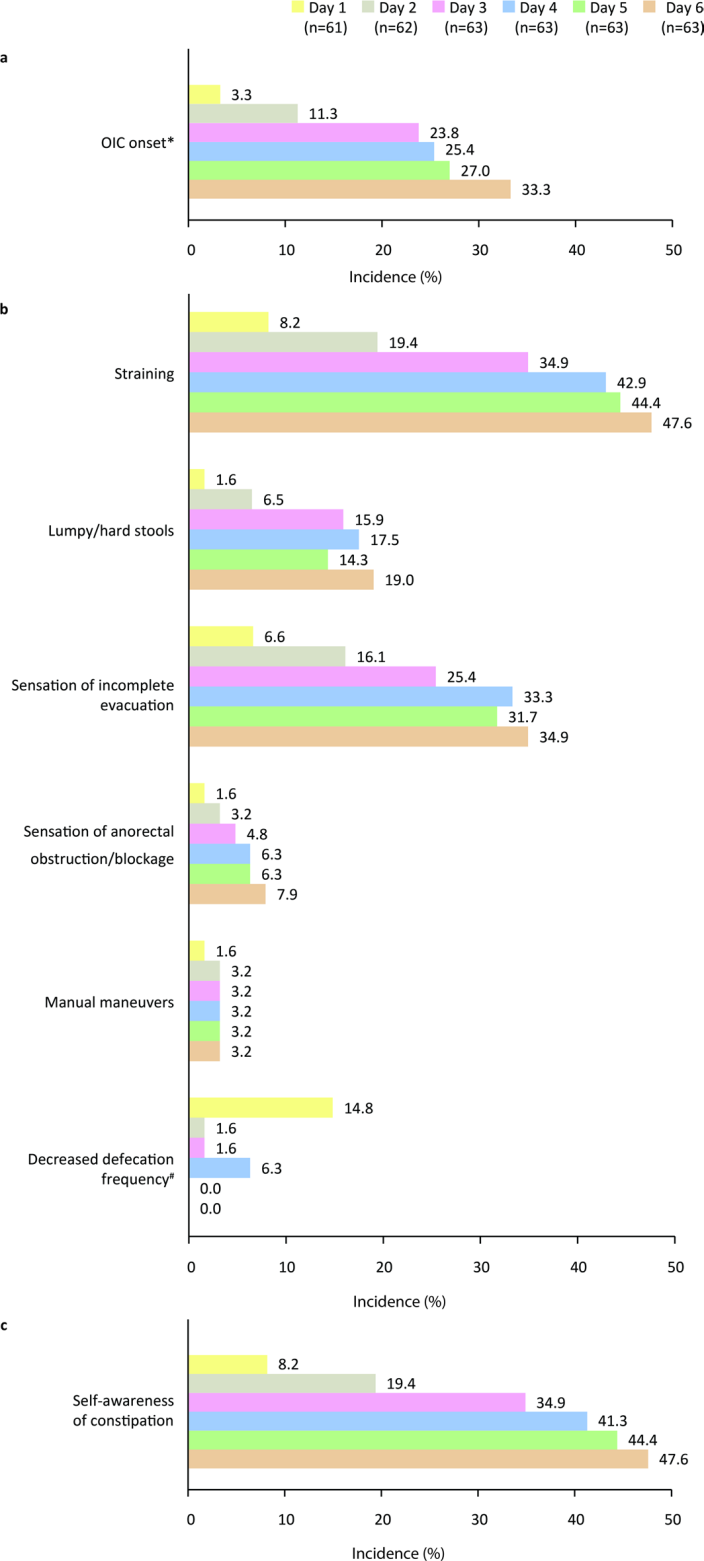
Incidence of (**a**) OIC onset, (**b**) constipation symptoms, and (**c**) self-awareness of constipation. ^*^Diagnosis based on Rome IV diagnostic criteria, which includes two or more symptoms of constipation. ^#^Defined as < 1 bowel movement (no defecation) from days 1 to 3 or < 2 bowel movements from days 4 to 6. Two and one patient did not respond on day 1 and day 2, respectively. OIC, opioid-induced constipation.

OIC onset was reported by 23.8% of patients at day 3 and 33.3% at day 6. The frequency and proportion of various constipation symptoms are shown in Fig. 1. At day 3, straining was the most common symptom, followed by sensation of incomplete evacuation and lumpy/hard stools (observed in 34.9%, 25.4%, and 15.9%, respectively). This progressively increased at day 6 (observed in 47.6%, 34.9%, and 19.0%, respectively). Sensation of anorectal obstruction/blockage, manual maneuvers, and decreased defecation frequency were each reported in < 5% of patients by day 3. Self-awareness of constipation paralleled straining and was seen in 34.9% at day 3; however, it exceeded the incidence of other constipation symptoms.

### Predictive accuracy of constipation symptoms and self-awareness of constipation

Sensitivity, specificity, LR+, LR-, and PPV of constipation symptoms are summarized in Table 2. As the specificity was 100%, the LR + for manual maneuvers and decreased defecation frequency was infinite from day 1 and day 2 onwards, respectively. The LR + for lumpy/hard stools and sensation of anorectal obstruction/blockage was 2.4 and 2.1 at day 3 and increased to 5.2 and 4.1 at day 6. The corresponding PPV were 70.0% and 66.7% at day 3 and 83.3% and 80.0% at day 6.

**Table 2:** Sensitivity, specificity, positive and negative likelihood ratios, and positive predictive values of various constipation symptoms.

Constipation symptoms		Day 1^*^ (*n* = 61)	Day 2^*^ (*n* = 62)	Day 3 (*n* = 63)	Day 4 (*n* = 63)	Day 5 (*n* = 63)	Day 6 (*n* = 63)
Straining	Number of cases (%)	5 (8.2)	12 (19.4)	22 (34.9)	27 (42.9)	28 (44.4)	30 (47.6)
	Number of cases with OIC on day 14	2	7	13	19	21	22
	Sensitivity, % (95% CI)	6.9 (0.8-22.8)	23.3 (9.9-42.3)	41.9 (24.5-60.9)	61.3 (42.2-78.2)	67.7 (48.6-83.3)	71.0 (52.0-85.8)
	Specificity, % (95% CI)	90.6 (75.0-98.0)	84.4 (67.2-94.7)	71.9 (53.3-86.3)	75.0 (56.6-88.5)	78.1 (60.0-90.7)	75.0 (56.6-88.5)
	LR+ (95% CI)	0.7 (0.2-2.8)	1.5 (0.8-2.9)	1.5 (0.9-2.4)	2.5 (1.7-3.5)	3.1 (2.3-4.2)	2.8 (2.1-3.8)
	LR- (95% CI)	1.0 (0.3-3.0)	0.9 (0.4-2.1)	0.8 (0.4-1.5)	0.5 (0.2-1.1)	0.4 (0.2-0.9)	0.4 (0.2-0.9)
	PPV (%)	40.0	58.3	59.1	70.4	75.0	73.3
Lumpy/hard stools	Number of cases (%)	1 (1.6)	4 (6.5)	10 (15.9)	11 (17.5)	9 (14.3)	12 (19.0)
	Number of cases with OIC on day 14	0	2	7	7	7	10
	Sensitivity, % (95% CI)	0.0 (0.0-11.9)	6.7 (0.8-22.1)	22.6 (9.6-41.1)	22.6 (9.6-41.1)	22.6 (9.6-41.1)	32.3 (16.7-51.4)
	Specificity, % (95% CI)	96.9 (83.8-99.9)	93.8 (79.2-99.2)	90.6 (75.0-98.0)	87.5 (71.0-96.5)	93.8 (79.2-99.2)	93.8 (79.2-99.2)
	LR+ (95% CI)	0.0 (-)	1.1 (0.3-4.1)	2.4 (1.2-4.7)	1.8 (0.9-3.5)	3.6 (1.9-7.0)	5.2 (3.1-8.7)
	LR- (95% CI)	1.0 (0.2-7.1)	1.0 (0.3-3.8)	0.9 (0.3-2.6)	0.9 (0.3-2.3)	0.8 (0.2-3.2)	0.7 (0.2-2.8)
	PPV (%)	0.0	50.0	70.0	63.6	77.8	83.3
Sensation of incomplete evacuation	Number of cases (%)	4 (6.6)	10 (16.1)	16 (25.4)	21 (33.3)	20 (31.7)	22 (34.9)
	Number of cases with OIC on day 14	2	5	9	14	15	16
	Sensitivity, % (95% CI)	6.9 (0.8-22.8)	16.7 (5.6-34.7)	29.0 (14.2-48.0)	45.2 (27.3-64.0)	48.4 (30.2-66.9)	51.6 (33.1-69.8)
	Specificity, % (95% CI)	93.8 (79.2-99.2)	84.4 (67.2-94.7)	78.1 (60.0-90.7)	78.1 (60.0-90.7)	84.4 (67.2-94.7)	81.3 (63.6-92.8)
	LR+ (95% CI)	1.1 (0.3-4.2)	1.1 (0.5-2.4)	1.3 (0.7-2.4)	2.1 (1.3-3.2)	3.1 (2.1-4.6)	2.8 (1.9-4.0)
	LR- (95% CI)	1.0 (0.3-3.8)	1.0 (0.4-2.2)	0.9 (0.5-1.8)	0.7 (0.3-1.5)	0.6 (0.3-1.5)	0.6 (0.3-1.3)
	PPV (%)	50.0	50.0	56.3	66.7	75.0	72.7
Sensation of anorectal obstruction/blockage	Number of cases (%)	1 (1.6)	2 (3.2)	3 (4.8)	4 (6.3)	4 (6.3)	5 (7.9)
	Number of cases with OIC on day 14	1	2	2	3	3	4
	Sensitivity, % (95% CI)	3.4 (0.1-17.8)	6.7 (0.8-22.1)	6.5 (0.8-21.4)	9.7 (2.0-25.8)	9.7 (2.0-25.8)	12.9 (3.6-29.8)
	Specificity, % (95% CI)	100.0 (89.1-100.0)	100.0 (89.1-100.0)	96.9 (83.8-99.9)	96.9 (83.8-99.9)	96.9 (83.8-99.9)	96.9 (83.8-99.9)
	LR+^#^ (95% CI)	Inf (-)	Inf (-)	2.1 (0.5-7.9)	3.1 (1.1-9.1)	3.1 (1.1-9.1)	4.1 (1.7-10.3)
	LR- (95% CI)	-	-	1.0 (0.1-6.7)	0.9 (0.1-6.4)	0.9 (0.1-6.4)	0.9 (0.1-6.2)
	PPV (%)	100.0	100.0	66.7	75.0	75.0	80.0
Manual maneuvers	Number of cases (%)	1 (1.6)	2 (3.2)	2 (3.2)	2 (3.2)	2 (3.2)	2 (3.2)
	Number of cases with OIC on day 14	1	2	2	2	2	2
	Sensitivity, % (95% CI)	3.4 (0.1-17.8)	6.7 (0.8-22.1)	6.5 (0.8-21.4)	6.5 (0.8-21.4)	6.5 (0.8-21.4)	6.5 (0.8-21.4)
	Specificity, % (95% CI)	100.0 (89.1-100.0)	100.0 (89.1-100.0)	100.0 (89.1-100.0)	100.0 (89.1-100.0)	100.0 (89.1-100.0)	100.0 (89.1-100.0)
	LR+^#^ (95% CI)	Inf (-)	Inf (-)	Inf (-)	Inf (-)	Inf (-)	Inf (-)
	LR- (95% CI)	-	-	-	-	-	-
	PPV (%)	100.0	100.0	100.0	100.0	100.0	100.0
Decreased defecation frequency^$^	Number of cases (%)	9 (14.8)	1 (1.6)	1 (1.6)	4 (6.3)	0 (0.0)	0 (0.0)
	Number of cases with OIC on day 14	6	1	1	4	0	0
	Sensitivity, % (95% CI)	20.7 (8.0-39.7)	3.3 (0.1-17.2)	3.2 (0.1-16.7)	12.9 (3.6-29.8)	-	-
	Specificity, % (95% CI)	90.6 (75.0-98.0)	100.0 (89.1-100.0)	100.0 (89.1-100.0)	100.0 (89.1-100.0)	-	-
	LR+^#^ (95% CI)	2.2 (1.1-4.5)	Inf (-)	Inf (-)	Inf (-)	-	-
	LR- (95% CI)	0.9 (0.3-2.6)	-	-	-	-	-
	PPV (%)	66.7	100.0	100.0	100.0	-	-

CI, confidence interval; Inf, infinity; LR-, negative likelihood ratio; LR+, positive likelihood ratio; OIC, opioid-induced constipation; PPV, positive predictive value.

*Two and one patient did not respond on day 1 and day 2, respectively.

^#^LR + was infinite due to the absence of false-positive cases.

^$^Defined as < 1 bowel movement (no defecation) from days 1 to 3 or < 2 bowel movements from days 4 to 6.

For self-awareness of constipation, LR+ was 2.8 at day 3 and 2.8 at day 6, with corresponding PPV of 72.7% and 73.3% (Table 3).

**Table 3:** Predictive accuracy of self-awareness of constipation.

Parameters	Day 1^*^ (*n* = 61)	Day 2^*^ (*n* = 62)	Day 3 (*n* = 63)	Day 4 (*n* = 63)	Day 5 (*n* = 63)	Day 6 (*n* = 63)
Number of cases (%)	5 (8.2)	12 (19.4)	22 (34.9)	26 (41.3)	28 (44.4)	30 (47.6)
Number of cases with OIC on day 14	3	7	16	19	21	22
Sensitivity, % (95% CI)	10.3 (2.2-27.4)	23.3 (9.9-42.3)	51.6 (33.1-69.8)	61.3 (42.2-78.2)	67.7 (48.6-83.3)	71.0 (52.0-85.8)
Specificity, % (95% CI)	93.8 (79.2-99.2)	84.4 (67.2-94.7)	81.3 (63.6-92.8)	78.1 (60.0-90.7)	78.1 (60.0-90.7)	75.0 (56.6-88.5)
LR+ (95% CI)	1.7 (0.6-4.9)	1.5 (0.8-2.9)	2.8 (1.9-4.0)	2.8 (2.0-3.9)	3.1 (2.3-4.2)	2.8 (2.1-3.8)
LR- (95% CI)	1.0 (0.2-3.7)	0.9 (0.4-2.1)	0.6 (0.3-1.3)	0.5 (0.2-1.1)	0.4 (0.2-0.9)	0.4 (0.2-0.9)
PPV (%)	60.0	58.3	72.7	73.1	75.0	73.3

CI, confidence interval; LR-, negative likelihood ratio; LR+, positive likelihood ratio; OIC, opioid-induced constipation; PPV, positive predictive value.

*Two and one patient did not respond on day 1 and day 2, respectively.

## Discussion

This post hoc analysis aimed to evaluate whether individual constipation symptoms could serve as early predictors of OIC, in patients taking weak opioids for non-cancer pain. Moreover, it assessed the predictive accuracy of these early constipation symptoms. Our findings showed OIC onset as early as 3 days after weak opioid initiation in 23.8% of patients. Straining, sensation of incomplete evacuation, and lumpy/hard stools were the most frequently observed symptoms at this time point. The proportion of patients reporting self-awareness of constipation was 34.9% at day 3, which is consistent with the early OIC onset. Manual maneuvers and decreased defecation frequency were observed in < 5% of patients. The PPV of developing OIC by day 14 was high when patients reported lumpy/hard stools and had self-awareness of constipation at day 3.

Our previous study reported cumulative OIC incidences of 30.2% on day 7 and 49.2% on day 14^11^; our present analysis showed that OIC onset had already occurred 3 days after weak opioid initiation. A randomized, placebo-controlled trial evaluated the influence of tramadol on bowel function and reported constipation symptoms in healthy participants. The study showed significantly decreased mean daily bowel movement on day 2 and increased colonic transit time on day 4 with tramadol^10^. Similar effects with strong opioids have been reported, including decreased colonic motility, delayed gastrointestinal transit time, and changes in the Bristol Stool Form scale within a week of opioid initiation^16–19^. Moreover, subjective symptoms of harder stool consistency were reported on day 4 in volunteers receiving weak opioids^20^. In addition, a prospective observational cohort study including 212 patients with cancer pain who were prescribed strong opioids, reported that self-recognition of constipation occurred in 30.2% of patients at day 3^21^. These findings highlight that OIC develops early within less than 1 week of opioid initiation. It suggests that identification of these symptoms can support early intervention and thereby improve patient outcomes and quality of care. In this study, patients who received prophylactic laxatives were excluded. However, during the observation period, the cumulative rate of patients starting treatment with laxatives was 31.3% at day 7 and 39.1% at day 14^11^. This suggests that, in this study population, intervention with laxatives was promptly initiated after the onset of OIC.

An ex vivo study in mice evaluated the effect of morphine on intestinal circular muscle contraction and compared the contractile waves across the jejunum, ileum, proximal, transverse, and distal colon, and rectum. It reported rapid initiation of circular muscle contractions in the rectum after opioid administration, suggesting that opioid-induced effects on gastrointestinal smooth muscle begin immediately^22^. Furthermore, randomized, double-blind, crossover trial in healthy males reported that opioids increase anal sphincter tone and decrease reflex relaxation, contributing to straining^19,23^. Since stools cannot be fully expelled, patients experience a sensation of incomplete evacuation^24^. Furthermore, opioids block inhibitory neural pathways via activation of µ-opioid receptors, which prolong intestinal transit and promote excessive water reabsorption, leading to the formation of lumpy/hard stools^17,25,26^. This may further increase straining and incomplete evacuation. Similar effects can be seen with weak opioid administration, which is likely due to the µ-opioid receptor-mediated mechanism, as these effects were also reported in non-clinical studies^9,27^, a randomized, placebo-controlled study^10^, and cohort studies^4,11^ in patients with non-cancer pain.

The present analysis also examined the predictability of early constipation symptoms for OIC onset by day 14 following weak opioid initiation. Among constipation symptoms, manual maneuvers, sensation of anorectal obstruction/blockage, and decreased defecation frequency in the first 6 days exhibited high specificity and LR+; however, the incidence of these symptoms was low. This suggests that when these symptoms are reported, it is reasonable to assume that OIC will develop regardless of the presence of other symptoms, even though they may result from more complex mechanisms. Symptoms such as lumpy/hard stools, straining, and sensation of incomplete evacuation by day 3 were more common and showed high predictive accuracies for the OIC development after weak opioid initiation. Our results are consistent with those of a previous population-based, cross-sectional survey in Spain reporting the highest accuracy of anal blockage, straining, and lumpy/hard stools for the diagnosis of constipation; however, it included subjects with chronic constipation^28^. In our study, few patients reported decreased defecation frequency, despite this often being used as a primary outcome in randomized controlled trials conducted in patients with OIC for cancer or non-cancer pain^29–32^. Symptoms of decreased defecation frequency would develop with longer duration of opioid treatments and remain an objectively measurable indicator of constipation severity in patients taking weak opioids. Additionally, in patients with OIC, the PPV of constipation symptoms based on Rome IV diagnosis is over 80%, with no notable differences in symptoms^15^. These observations suggest the importance of considering the duration since opioid initiation when assessing symptoms.

In addition to OIC onset and constipation symptoms defined by the Rome IV diagnostic criteria, this analysis investigated the potential to predict OIC onset based on self-awareness of constipation. The incidence of self-awareness of constipation was higher than that of any individual constipation symptom. Similarly, a cross-sectional survey among 489 patients with chronic constipation (rather than OIC) reported that self-perception of constipation was greater than constipation determined by objective criteria^28^. Based on this, we speculate that self-awareness of constipation in patients initiating weak opioids could potentially predict the onset of OIC. Our findings at day 3 of opioid use showed that the LR+ and PPV for self-awareness of constipation surpassed the predictive accuracy of straining, sensation of incomplete evacuation, and lumpy/hard stools. These suggest that, in clinical practice, simply assessing patients’ self-awareness of constipation could enhance predictive accuracy for OIC onset, besides querying specific constipation symptoms. Crucially, although OIC is a common side effect of opioid therapy, early assessment of constipation symptoms and self-awareness of constipation soon after initiating opioid use could potentially predict the onset of OIC with high accuracy. Physicians should ensure that opioid treatment is appropriate for the patients when initiating opioids. They should also confirm that patients are enrolled in a pain management program, have received the minimum effective opioid dose, and have been provided appropriate education regarding OIC at the start of opioid therapy, in accordance with clinical guidelines^1,2,33^.

This study has several limitations intrinsic to the study design, as the findings are based on a post hoc analysis rather than a planned analysis. First, the diagnosis of OIC was based solely on self-reported patient outcomes without physician confirmation. In addition, the location of pain was patient-reported, and neither a clinical diagnosis of pain nor its presumed etiology was available, which may introduce reporting bias. Second, the relatively small sample size may limit the precision of the findings. Third, the onset of OIC was defined according to the Rome IV diagnostic criteria with modification, which have not been validated for diagnosing OIC in the early stages (within less than 1 week of opioid initiation); this may introduce bias into diagnostic accuracy. Furthermore, some patients took laxatives after starting opioid therapy, which could have reduced symptoms before the full diagnostic criteria were met, potentially leading to an underestimation of OIC incidence. Lastly, patients were informed in advance about the potential development of OIC, which might have increased the proportion of patients experiencing OIC or decreased it due to modifications in their dietary habits, such as increasing dietary fiber intake.

## Conclusion

This study demonstrates that OIC develops shortly, within 1-3 days after initiation of weak opioids in non-cancer patients. The early constipation symptoms, such as lumpy/hard stools and straining, and the patients’ self-awareness of constipation are crucial for accurately predicting OIC onset. These findings highlight the importance of early monitoring of constipation symptoms during the first week of opioid initiation to prevent complications and enhance patient comfort.

## Data Availability

The datasets generated and/or analyzed during the current study are available from the corresponding author upon reasonable request.
